# Pharmacological Inhibition of Serine Proteases to Reduce Cardiac Inflammation and Fibrosis in Atrial Fibrillation

**DOI:** 10.3389/fphar.2019.01420

**Published:** 2019-12-20

**Authors:** Raffaele Coppini, Lorenzo Santini, Chiara Palandri, Laura Sartiani, Elisabetta Cerbai, Laura Raimondi

**Affiliations:** Section of Pharmacology, Department of Neurology, Psychology, Drug Sciences and Child Health, University of Florence, Florence, Italy

**Keywords:** atrial fibrillation, coagulation, fibrosis, inflammation, protease-activated receptors

## Abstract

Systemic inflammation correlates with an increased risk of atrial fibrillation (AF) and thrombogenesis. Systemic inflammation alters vessel permeability, allowing inflammatory and immune cell migration toward target organs, including the heart. Among inflammatory cells infiltrating the atria, macrophages and mast cell have recently attracted the interest of basic researchers due to the pathogenic mechanisms triggered by their activation. This chemotactic invasion is likely implicated in short- and long-term changes in cardiac cell-to-cell communication and in triggering fibrous tissue accumulation in the atrial myocardium and electrophysiological re-arrangements of atrial cardiomyocytes, thus favoring the onset and progression of AF. Serine proteases are a large and heterogeneous class of proteases involved in several processes that are important for cardiac function and are involved in cardiac diseases, such as (i) coagulation, (ii) fibrinolysis, (iii) extracellular matrix degradation, (iv) activation of receptors (i.e., protease-activated receptors [PPARs]), and (v) modulation of the activity of endogenous signals. The recognition of serine proteases substrates and their involvement in inflammatory/profibrotic mechanisms allowed the identification of novel cardio-protective mechanisms for commonly used drugs that inhibit serine proteases. The aim of this review is to summarize knowledge on the role of inflammation and fibrosis as determinants of AF. Moreover, we will recapitulate current findings on the role of serine proteases in the pathogenesis of AF and the possible beneficial effects of drugs inhibiting serine proteases in reducing the risk of AF through decrease of cardiac inflammation and fibrosis. These drugs include thrombin and factor Xa inhibitors (used as oral anticoagulants), dipeptidyl-peptidase 4 (DPP4) inhibitors, used for type-2 diabetes, as well as novel experimental inhibitors of mast cell chymases.

**Graphical Abstract:**
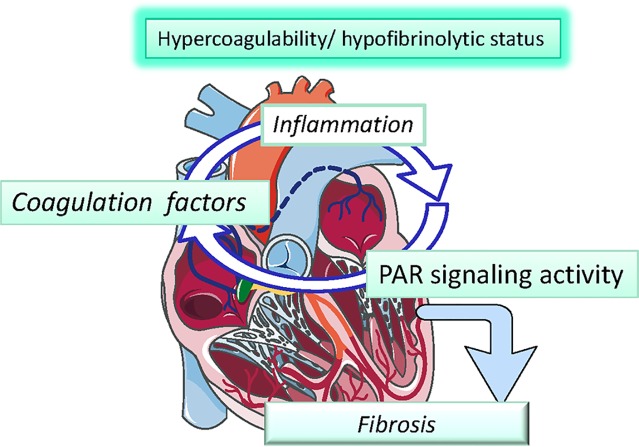
Inflamation and Hypercoagulability.

## Introduction

Atrial fibrillation (AF) is the most frequent cardiac arrhythmia in the clinical practice (Karnik, 2019). The prevalence of AF is rapidly growing in Europe and in the US, as it closely follows the rapidly increasing prevalence of heart failure in the aging population (Vasan et al., 2019). However, while total mortality for heart failure has remained stable in the last few years, due to treatment improvement and optimization (Vasan et al., 2019), hospitalization rates and mortality for AF are steadily rising (Freeman, 2017). Indeed, AF is associated with an increased risk of heart failure decompensation, as well as to arterial thromboembolism leading to ischemic stroke (Best, 2019; Carlisle, 2019). AF is, therefore, a major health issue, associated with significant social and economic costs in developed countries. The main risk factors for AF are hypertension, valvular disease, cardiomyopathies, heart failure with reduced or preserved ejection fraction, obesity, and diabetes. Most of these AF predisposing conditions are associated with systemic inflammation and cardiac fibrosis, two major pathophysiological determinants of the functional and structural abnormalities of atrial myocardium that precede the onset of atrial arrhythmias.

In the first part of the review, we will focus on the role of local and systemic inflammation as a risk factor for the development and progression of AF, focusing on the cellular and molecular mechanisms linking inflammation to atrial electrical abnormalities. We will then describe the role of atrial fibrosis as a pathological mechanism predisposing to distinct AF-linked electrical abnormalities, through alteration of both atrial excitability and conduction by proliferation of collagen and fibroblasts. Afterward, we will summarize recent work on the role of coagulation factor serine proteases (thrombin and factor Xa) and protease-activated receptors (PARs) as determinants of atrial fibrosis, inflammation, and AF-related electrical abnormalities; with this regard, we will describe how novel anticoagulants, through inhibition of thrombin or factor Xa, may affect the pathophysiology of AF and alter disease progression. Finally, we will discuss the possible role of other serine proteases active in the atria (mast-cell chymases and dipeptidyl-peptidase 4) and the possible efficacy of their selective inhibitors in modulating the risk of AF.

## AF and Inflammation

The occurrence and perpetration of AF is usually preceded by structural modifications of atrial structure and function (atrial remodeling), both at macroscopic level and at cellular/subcellular scale. At macroscopic level, atrial dilatation and loss of atrial contractility often precede AF, paralleled by proliferation of fibroblasts and collagen (fibrosis) in atrial myocardium (Greiser, 2009; Jalife and Kaur, 2015; Rapacciuolo, 2019). At cellular/subcellular level, arrhythmia susceptibility is caused by changes of the expression and function of ion channels leading to alterations of L-type calcium current, calcium-activated, and acetylcholine-activated potassium currents, as well as changes of ryanodine receptors leading to alterations of intracellular calcium handling (Neuberger, 2006; Denham, 2018). Hypertension, valvular heart disease, and cardiomyopathies can initiate atrial dilatation and remodeling due to the increased left ventricular filling pressures leading to increased atrial pressure and wall stress: as atrial muscle is relatively thin and atria are low-pressure chambers, they respond to increased pressures with dilatation rather than wall thickening and hypertrophy (Nattel and Harada, 2014). In addition to the hemodynamic changes associated with the aforementioned conditions, the pathophysiological mechanism linking predisposing conditions to persistent AF involves changes in several molecular pathways within atrial cardiomyocytes and all the surrounding myocardial cell types and structures (e.g., endothelial cells and small vessels, fibroblasts, and extracellular matrix, epicardial fat tissue) (Heijman, 2016).

### Systemic Inflammation Is Associated With AF

Evidence suggests that inflammatory and oxidative pathways may mediate, at least in part, the progression of atrial remodeling, and may contribute to the initiation of AF and to progression of the arrhythmia from paroxysmal/episodic to persistent/permanent. Interestingly, most cardiovascular and systemic conditions that are associated with AF are also associated with low-grade chronic inflammation (Hu, 2015). These conditions include heart failure, arterial hypertension, coronary artery disease (CAD), obesity, sleep-apnea syndrome, and chronic obstructive pulmonary disease (COPD), as well as diabetes mellitus (Andrade, 2014). Angiotensin II, one of the most important mediators of vascular dysregulation in hypertension, on top of vasoconstriction, also induces production of pro-inflammatory cytokines, and activation of immune cells. Heart failure, the main condition predisposing to AF, is characterized by cardiac inflammation and increased immune activation within the ventricular myocardium, contributing to cardiomyocyte death, myocardial fibrosis, and mechanical dysfunction (Ayoub, 2017). In CAD, progression of atherosclerosis is driven by chronic inflammation of blood vessels. In these conditions, inflammation may affect atrial muscle and promote AF (da Silva, 2017). Interestingly, it has been observed that typical markers of atherosclerosis such as coronary artery calcifications can also predict the risk of AF (da Silva, 2017). In COPD, generalized inflammation and hypoxia-linked oxidative stress, paralleled by alterations of the autonomic balance, may promote AF in the absence of heart failure or hypertension (Goudis, 2017). In patients with AF, the onset of COPD is associated with increased AF recurrences, low likelihood of cardioversion, faster progression to permanent arrhythmia (Goudis, 2017). It is to be noted that the main risk factor for COPD, that is cigarette smoking, is linked with oxidative damage in the lungs and in the cardiovascular system, and is a known promoter of inflammation (Zhu, 2016). Diabetes mellitus is also linked with systemic immune activation and chronic generalized inflammation. Moreover, hyperglycemia may have direct deleterious effects on atrial cardiomyocytes, promoting electrical and mechanical abnormalities that predispose to atrial remodeling and AF (Goudis, 2015). Obesity increases the likelihood of AF with several mechanisms (Lavie, 2017): 1) increases the risk of hypertension, diabetes, and CAD; 2) promotes the growth of epicardial fat, which is linked to local inflammation; 3) is often associated with obstructive sleep apnea (OSA) syndrome. OSA favors atrial volume overload, cause hypoxia and oxidative stress, and is linked with systemic inflammation, thus representing an independent risk factor for AF (Goudis and Ketikoglou, 2017).

### Local Inflammation Is a Cause of AF

The effects of local inflammation on atrial function have been studied in cardiac surgery patients (Bruins, 1997). Cardiac operations, especially those requiring pericardiotomy and atriotomy, lead to acute inflammation of the pericardium and myocardium: between the second and the fourth day after surgery, post-operative AF may occur in surgical patients, as a direct consequence of the inflammatory processes that follow the operation (Maesen, 2012). Post-operative AF is usually short lasting and does not evolve into chronic arrhythmia, rather, it tends to disappear as local wounds repair and local inflammation levels decrease. Nonetheless, it may complicate the recovery from cardiac surgery due to the heightened risk of thromboembolism and the deleterious hemodynamic consequences, thus requiring aggressive treatment. Another local inflammatory condition associated with AF is gastroesophageal reflux with active esophagitis: lower esophagus is in close proximity with the atria and esophageal inflammation may also affect the neighboring atrial muscle, thus promoting AF (Linz, 2017).

### How Does Inflammation Promote AF?

It is difficult to separate the direct deleterious effects on atrial muscle of conditions associated with AF (e.g., hypertension, diabetes or CAD) from those mediated only by systemic inflammation, which is always present in these conditions. Therefore, the direct effects of inflammation on the atria have been studied in purely inflammatory diseases such as autoimmune rheumatic syndromes (Baek, 2016), where the risk of AF is increased in the absence of any local or hemodynamic conditions that damage the atria. In rheumatic diseases, the increase of circulating inflammatory mediators is directly associated with dysregulation of connexins, leading to altered gap junction function and uneven electrical conduction (Lazzerini et al., 2017). Moreover, cytokines have been shown to induce acute abnormalities of intracellular calcium handling, leading to cardiomyocyte calcium overload and to an increased rate of Ca^2+^-dependent arrhythmias (Korantzopoulos, 2003; Hu, 2015). A number of inflammatory mediators have been implicated in the generation of pro-arrhythmic changes in the atria. In particular, interleukin (IL)-2 and tumor necrosis factor alpha (TNFα) impair calcium handling and leads to arrhythmogenic electrophysiological changes (e.g., shortening of action potentials) in atrial cardiomyocytes, in particular in the region adjacent to pulmonary veins (PVs) in the left atrial posterior wall, a hot-spot for atrial arrhythmias (Korantzopoulos, 2003; Hu, 2015). High levels of TNFα in the mouse lead to reduced systolic calcium transient amplitude and elevated diastolic calcium concentration within atrial cardiomyocytes (Saba, 2005). Ca^2+^ transients are also prolonged, due to a reduction of sarcoplasmic endoplasmic reticulum calcium ATPase (SERCA) expression, an increase of Na^+^/Ca^2+^ exchanger (NCX) activity, and a decrease of phospholamban. Altered TNFα-mediated Ca^2+^ handling could lead to arrhythmias due to calcium waves and delayed after-depolarization, which in turn may generate premature APs and give birth to spontaneous beats that can then perpetuate themselves through re-entry. In addition to promoting atrial cellular arrhythmias, circulating cytokines can also stimulate NF-κB within atrial cardiomyocytes, leading to activation of the apoptosis cascade, which in turn is responsible for cardiomyocyte death and fibrous substitution.

### AF Causes Inflammation: Role of Infiltrating Inflammatory Cells

While inflammation can cause AF, AF can cause both local and generalized inflammation (Korantzopoulos, 2018). In patients with isolated forms of AF (“lone” AF), who do not have any other underlying cardiac or extracardiac AF-associated disease, atrial tissue displays a mild level of local inflammation comprising cellular infiltration (Frustaci, 1997), which appears to be a direct consequence of the arrhythmia. Indeed, atrial tachycardia causes intracellular calcium overload, which in turn leads to energetic insufficiency and oxidative stress, and may stimulate apoptosis (Van Wagoner, 2008; Hu, 2015). These processes lead to activation of resident immune cells and production of cytokines, promoting local inflammation. In line with that, it was shown that C-reactive protein and IL-6, two circulating mediators of inflammation, are constantly elevated in patients during and shortly after episodes of AF, even in patients with “lone” AF (Marcus, 2010). Blood levels of C-reactive protein and IL-6 in patients with AF are inversely related with the success rate of cardioversion (Marcus, 2010). Other acute-phase circulating inflammatory proteins, such as fibrinogen, were associated with AF: in patients who received a successful electrical cardioversion, circulating fibrinogen levels paralleled the risk of AF recurrence (Weymann, 2017). Markers of immune cell activation are also related with the risk of AF: an increased white blood cell count was associated with the incidence of AF. In particular, the combination of a high white cell count with an elevated neutrophil-to-lymphocyte ratio has an elevated predictive power for AF recurrences (Im, 2013; Shao, 2015). In patients with paroxysmal “lone” AF, a study found an elevated count of circulating neutrophils and eosinophils: eosinophil count was independently linked with the probability of AF relapses (Chen, 2017). The risk of AF progression from paroxysmal to persistent arrhythmia appears also to be associated with the recruitment of macrophages in the atrial endocardium, supporting the idea that local inflammatory activation contributes to arrhythmia progression and worsening (Yamashita, 2010). This macrophagic infiltrate produces reactive oxygen species, as well as high levels of TNF, transforming growth factor (TGFβ), and IL-6 (Pinho-Gomes, 2014). Interestingly, in patients with rheumatic mitral stenosis, proinflammatory M1 macrophages infiltrating atrial tissue were much more abundant in patients with a history of AF, with respect to patients without atrial arrhythmias (He, 2016), suggesting the key role of infiltrating macrophages in mediating local atrial inflammation in AF. An elegant study evidenced a clear cross-talk in vitro between macrophages and atrial cardiomyocytes. On one hand, fast stimulation of atrial myocytes (simulating AF) led to pro-inflammatory macrophage polarization. On the other hand, activation of macrophages with lipopolysaccharide (LPS) led to reduced Ca^2+^ current and refractory period, leading to increased arrhythmic events, in atrial myocytes (Sun, 2016). The activation of resident inflammatory cells by pro-inflammatory mediators causes local oxidative cellular damage mediated by myeloperoxidase (MPO), as well as disruption of atrial tissue organization by secreted proteases (see below). Indeed, MPO is a catalytic enzyme that generates reactive oxygen species, affecting several signaling cascades in cardiomyocytes and other cell types (Friedrichs et al., 2012). Circulating MPO levels are higher in patients with AF, and MPO is particularly concentrated in atrial tissue in AF patients. Of note, MPO stimulates local production of matrix-metalloproteinases (MMPs), which contribute to tissue remodeling and fibrous substitution. These clinical and preclinical studies support the idea that AF episodes can generate an inflammatory state that in turn stimulates the formation of stable pro-arrhythmic structural abnormalities in the atria, leading to chronic/persistent arrhythmia.

### Role of Mast Cells in the Pathogenesis of AF

In addition to infiltrating macrophages and neutrophils, resident atrial mast cells were also found to be involved in AF pathogenesis. In patient with AF and thrombosis in the atrial appendages, the number of mast cells and the expression of mast-cell growth factor were markedly increased in atrial tissue, with respect to patients without AF or thrombosis (Bankl, 1995). In an animal model of pressure overload, mast cells accumulated in the dilated atria: activated mast cells produced a large amount of platelet-derived growth factor (PDGF)α, which in turn promoted fibroblast activation and fibrosis (see below). Interestingly, neutralizing PDGFα-receptor with specific antibody attenuated atrial fibrosis and inducibility of AF in the atria of pressure-overloaded animals (Liao, 2010). In a model of streptozotocin-induced diabetes in the mouse, hyperglycemia led to increased mast-cell infiltration in the atria, paralleled by atrial fibrosis, cardiomyocyte apoptosis, and increased production of cytokines. In transgenic mast-cell deficient mice, where mast cell infiltration was prevented, most of the aforementioned pathological changes were greatly attenuated (Uemura, 2016). Activated mast cells produce mast cell chymases, serine proteases with possible pro-arrhtythmic effects in the atria (see below).

### Effects of Anti-Inflammatory Drugs on the Risk of AF

A confirmation of the important role of inflammation in the pathophysiology of AF comes from studies assessing the effects of pharmacological agents with anti-inflammatory activity in patients at risk of AF. A meta-analysis of 42 small randomized placebo-controlled studies (over 4500 total patients) confirmed that glucocorticoid prophylaxis in patients who underwent cardiac surgery reduced the risk of perioperative AF (Liu, 2014). However, a larger carefully conducted study showed that the risk of perioperative AF was 33% in patients who received dexamethasone and 35% in patients who received placebo, showing no clear advantage of corticosteroid treatment in this clinical setting (van Osch, 2015). In patents who underwent PV trans-catheter radiofrequency ablation for AF, instead, a few days of dexamethasone treatment decrease the risk of short-term relapses (Kim, 2015). Another anti-inflammatory and anti-chemotactic agent that was found effective in preventing post-operative and post-ablation AF is colchicine, an inhibitor of microtubule polymerization (Salih, 2017). Ablation is a procedure that generates programmed damage to atrial myocardium, aiming to block electrical conduction from arrhythmic hot-spots like PVs. Tissue damage generates local edema and inflammation, which may be the primary cause of early AF relapses (Lim, 2014); hence the advantage of using anti-inflammatory medications after ablation. Drugs that are used for the reduction of cardiovascular risk factors, such as statins, may have an additional anti-inflammatory efficacy, which may be of benefit for the prevention of AF. Pre-treatment with statins reduces the risk of recurrences after electrical cardioversion and decreases the likelihood of AF after cardiac surgery (Zheng, 2016; An, 2017). This effect appears to be linked with the anti-inflammatory actions of statins (Pinho-Gomes, 2014), as statin-treatment it is associated with a reduced peak of circulating C-reactive protein after surgery. Finally, aldosterone antagonists may help preventing AF due to their anti-inflammatory properties, on top of their anti-fibrotic and anti-oxidant capabilities (Korantzopoulos and Goudevenos, 2010). Finally, treatment with mineralocorticoid receptor blockers was also shown to be associated with a reduced incidence of new-onset AF and a lower frequency of recurrent events (Neefs, 2017).

## AF and Fibrosis

### Pathophysiology of Atrial Fibrosis

In all tissues and organs, the fibrotic process involves an increased activity of fibroblasts and an amplified production of various components of the extracellular matrix (ECM) by them (Nattel, 2017). These include fibronectin (which forms an initial scaffold for fibroblast to attach and collagen fibers to build up), procollagen (later converted to mature fibrous collagen thanks to cross-linkers), enzymes that cross-link collagen (e.g., lysyl-oxidase), enzymes that are able to modify and remodel the ECM, such as MMPs, as well as inhibitors of MMPs. Fibroblasts are small spindle-like cells constituting about 10-15% of total cardiac mass (Yue et al., 2011), both in atria and in ventricles. Cardiomyocytes are approximately 70% to 75% of total myocardial mass in the ventricles, while only about 45% in the atria (Hinescu and Popescu, 2005). The physiological amount of ECM in the atria is therefore much higher in the atria than in the ventricles; this is probably the result of the different phenotype and activity of atrial fibroblasts as compared with the ventricular counterpart (Hinescu, 2006). Fibroblasts are continuously active in maintaining the integrity of cardiac ECM, modifying its structure and composition in response to physiological and pathological stimuli. For this reason, fibroblasts communicate with the surrounding environment via direct connection with other cell types or with matrix components, or via a number of paracrine mediators, growth factors, and cytokines (Camelliti et al., 2005). In response to cardiac tissue damage, fibroblasts migrate to the damage site and become activated by differentiating into a different cell type that resemble small muscle (the so called “myofibroblasts”) (Majno, 1971). Myofibroblast phenotype changes from proliferative to secretory and they start producing compact fibrous collagen matrix, aiming to repair the damage and rapidly create a scar (Cleutjens, 1995).

### Evidence That AF Is Associated With Atrial Fibrosis

The first work that showed a causative association between atrial fibrosis and AF was published in 1999 by the group of Stanley Nattel (Li, 1999; Nattel, 2016): in a canine model of congestive heart failure, progressive development of atrial fibrosis went hand-in-hand with the increased susceptibility to atrial arrhythmia induction. Mathematical models simulating the role of electrical conduction blocks in cardiac tissue had previously suggested a possible role for fibrosis in the pathogenesis of AF (Spach and Josephson, 1994). Moreover, it had been observed that the atria from patients with AF displayed increased myocardial fibrosis (Frustaci, 1997). Indeed, atrial fibrosis is a very common finding in human AF (Kostin, 2002). Increased collagen has been shown also in patients with “lone” AF (Frustaci, 1997). In general, the degree of ECM expansion correlates with the persistence of AF (Xu, 2004). AF is correlated with an increased expression of pro-fibrotic genes and proteins (see below) in human atrial biopsies (Barth, 2005). The link between fibrosis and atrial arrhythmia was supported by the observation that fibrosis and the resulting abnormalities of cardiac conduction promote initiation and maintenance of AF even in the absence of abnormalities of atrial cardiomyocyte ion currents (Cha, 2004).

### How Atrial Fibrosis Causes Electrical Abnormalities

The reparative (replacement) fibrosis is aimed at substituting regions of dead cardiomyocytes with fibrous tissue (Weber, 2013): in the atria of animal models of heart failure, increased atrial filling pressures and atrial dilatation causes the formation of extended regions of cell death in the atria. Dead cardiomyocytes are replaced by collagen, thus creating regions of fibrosis that are intercalated among surviving muscle bundles and represent a clear barrier to electrical conduction in the longitudinal direction (Hanna, 2004). These new ECM deposits interfere with the formation of connexin-rich tight junctions (gap junctions) and can cause slowing of electrical conduction or even local conduction blocks (Burstein, 2009). Reactive fibrosis, instead, occurs mainly in response to local of systemic inflammatory stimuli in the absence of an extensive myocyte death: activated myofibroblast produce collagen strands that accumulate in the space surrounding blood vessels and cardiomyocytes, creating thick sheaths that surround myocardial fibers, thereby insulating them and impeding transversal conduction (Krul, 2015). The insulation of myocardial fibers in the presence of reactive interstitial fibrosis accelerate longitudinal conduction within the fiber, favoring the formation of multiple reentry circuits (Burstein and Nattel, 2008).

### Active Role of Fibroblasts in Atrial Electrical Dysfunction

In addition to the consequences of the increased collagen, activated fibroblasts can directly modify atrial electrical activity due to formation of gap junctions with cardiomyocytes (Camelliti, 2004). Because membrane potential of fibroblast is less negative that that of cardiomyocytes, this electrotonic interaction causes the depolarization of all myocytes forming gap junctions with fibroblast, ultimately resulting in slower conduction speed due to reduced sodium channel availability (Miragoli et al., 2006). Myocyte depolarization favors spontaneous activity (Miragoli et al., 2007), while areas of slower conduction may favor reentry. Studies in co-cultures of fibroblasts and myocytes, as well as mathematical models, suggest that electrical interaction between fibroblasts and myocytes may significantly alter the properties of AF (Aguilar, 2014), on top of the consequences of fibrosis. In a recent modeling study (Zahid, 2016), the atria of AF patients were reconstructed in silico from detailed magnetic resonance images providing information on the distribution of fibrosis (gadolinium enhancement). By simulating the electrical activity of the atria in the presence of fibrosis, the model showed that triggers for atrial arrhythmias are generated in areas of high coupling between myofibroblasts and cardiomyocytes, and the consequent ectopic activation can generate stable reentry circuits where electrically active myocytes surround larger fibrotic regions, that constitute anchors for reentry (Schotten, 2011).

### Role of Ion Channels Expressed on Fibroblast Membrane

The relationship of cardiomyocytes with fibroblasts in the atria is made more complicated by the observation that fibroblasts express some ion channels (Yue et al., 2011). Cardiac fibroblasts cannot be defined as electrically excitable cells, however they show a resting membrane potentials (RMPs) with average values of −37 mV (Kamkin et al., 2003), whose primary determinant is represented by the inward-rectifier K^+^ current, I_K1_ (Chilton, 2005)_._ Qi et al. evaluated the potential effects of fibroblast I_K1_ modulation in a canine model of heart failure, noticing that fibroblast I_K1_ upregulation promoted fibroblast proliferation and the development of pro-arrhythmic atrial structural changes, while downregulation of fibroblasts I_K1_ lead to suppression of atrial fibrosis and arrhythmogenesis (Qi, 2015). The potential influence of ﬁbroblast I_K1_ currents on cardiac electrical activity was recently evaluated by Aguilar et al. through a mathematical model of cardiomyocyte-ﬁbroblast coupling (Aguilar, 2014). The authors of this study showed that upregulation of I_K1_ currents in ﬁbroblasts has proﬁbrillatory consequences, principally caused by the shortening of atrial action potential duration. The functional role of cardiac fibroblasts has also been evaluated in studies conducted on human samples. Poulet et al. compared atrial ﬁbroblasts from patients in sinus rhythm (SR) to that of patients affected by chronic AF and found that AF ﬁbroblasts differentiated into myoﬁbroblasts more readily than SR fibroblasts and were characterized by larger Na^+^ and I_K1_ currents, compared to currents recorded in the SR group. This was accompanied by changes in electrophysiological properties which may contribute to the pathophysiology of AF(Poulet, 2016).

### Signaling Cascades Involved in the Generation of Atrial Fibrosis in AF

#### The Renin-Angiotensin System

Angiotensin II is produced by cardiomyocytes upon stretching (Malhotra, 1999) and can directly affect fibroblasts and cause their activation. Several studies have highlighted that myocardial fibrosis in heart failure (Weber et al., 1993), myocardial infarction (Hanatani, 1995), and cardiomyopathies is, at least in part, driven by the activation of the renin-angiotensin system. The role played by the renin-angiotensin system in the development of atrial fibrosis has been investigated in different animal models. Indeed, angiotensin-converting enzyme (ACE) overexpression, induced in a transgenic mouse model, was responsible for atrial fibrosis (Xiao, 2004). Moreover, many studies have shown the ability of ACE inhibitors to significantly reduce the occurrence of AF in animal models and patients (Shi, 2002; Healey, 2005). Similar results have been observed in canine models with either experimental heart failure or rapid atrial pacing, where enalapril was shown to reduce atrial fibrosis and the duration of AF episodes (Sakabe, 2004). Moreover, candesartan (angiotensin II type 1 receptor blocker) was capable to prevent both fibrosis and atrial structural remodeling in rats (Okazaki, 2006) and dogs (Kumagai, 2003). In patients with AF and atrial fibrosis, Goette et al. observed enhanced ERK activation in association to increased Angiotensin II concentration in atrial tissue (Goette, 2000). Retrospective clinical studies show that the incidence of AF is decreased in patients treated with angiotensin-receptor blockers (ARBs) or ACE inhibitors (Vermes, 2003; L’Allier, 2004). Taken together, clinical data support the use of ACE inhibitors to delay the onset and progression of atrial fibrosis and AF. Recent studies showed that the fixed combination of an ARB (valsartan) with a neprilysin inhibitor (sacubitril) was able to reduce cardiac fibrosis and the risk of ventricular arrhythmias and sudden death in patients with heart failure (Sarrias and Bayes-Genis, 2018; Aimo et al., 2019; Martens, 2019). However, the valsartan/sacubitril combination does not appear to have additional beneficial effects on atrial arrhythmic burden, as compared with ARBs or ACE-I alone (Martens, 2019). Neprilysin catalyzes the degradation of natriuretic peptides (atrial- and brain-derived natriuretic peptides, ANP, and BNP, respectively), which have anti-fibrotic effects. As clinical studies showed that neprilysin inhibition has antifibrotic and antiarrhythmic effects only in the ventricles, the downstream signaling pathway of natriuretic peptides may be scarcely relevant for the regulation fibrous tissue in the atria.

#### Transforming Growth Factor β1

TGFβ1 stimulates collagen production exerting its effect through the “small mother against decapentaplegic” (SMAD) signaling pathway (Evans, 2003). Studies have highlighted that TGFβ1 expression cause an increase of myocardial fibrosis (Lijnen et al., 2000). In fact, selective atrial interstitial fibrosis was evidenced in a transgenic mouse model characterized by the overexpression of a constitutively active form of TGFβ1. Albeit the overexpression of TGFβ1 was comparable in the atrium and the ventricles, abnormalities were evidenced only in atrial tissue, suggesting that the atria are more susceptible than the ventricles to the development of TGFβ1-induced fibrosis (Verheule, 2004). Atrial TGF-β1 is released in response to hypertrophic stimuli and prolonged stretch by multiple cell types, including cardiomyocytes, fibroblasts, and endothelial cells; these cells also express TGF-β1 receptors, so its signaling is both paracrine and autocrine (Nattel, 2017). The augmentation of atrial fibrosis was associated with increased AF vulnerability and conduction abnormalities. In an experimental model of heart failure, characterized by an increased TGF-β1 expression and atrial fibrosis, the authors showed that the administration of the drug pirfenidone (a novel antifibrotic agent in use for idiopathic pulmonary fibrosis) was able to significantly reduce the expression of TGFβ1, with a concomitant decrease in atrial fibrosis, conduction abnormalities, and AF vulnerability (Lee, 2006). Interestingly, a large placebo-controlled study with pirfenidone is ongoing in patients with chronic heart failure and preserved ejection fraction (HFpEF), a condition that is strongly associated with the risk of AF (Lewis, 2019).

#### The Oxidative Stress Pathways

Oxidative stress is likely to play a leading role in promoting AF, as evidences of oxidative damages are observed during AF episodes (Mihm, 2001). The oxidative stress could represent an important starting point for the development of AF through the activation of the ERK intracellular pathway.

#### Platelet-Derived Growth Factor

PDGF is a cytokine that stimulates fibroblast proliferation and their subsequent differentiation to myofibroblasts (Fan, 2014) through the direct activation of the Janus kinase (JAK)-STAT pathway (Patel, 1996). PDGF-β is prominently produced by activated resident mast cells within affected atria, thus linking fibrosis with inflammation. In the heart, the activation of the JAK-STAT pathway by PDGF-β stimulates cardiomyocyte survival and simultaneously reduces apoptosis by positively regulating the expression of several cardioprotective and anti-inflammatory-related genes (Kishore and Verma, 2012) (Jacoby, 2003). The potential ability of PDGF-JAK-STAT system to modulate the behavior of atrial fibroblast and atrial-selective fibrosis was evaluated by Chen et al. (Chen, 2017) on dogs with heart-failure caused by prolonged ventricular tachypacing. The authors of this study observed that PDGF-mediated stimulation of atrial fibroblasts triggered an up-regulation of JAK-STAT expression and its consequent increased activity was associated to an augmented production of ECM-protein. This study also showed that AG 1296 (PDGF receptor inhibitor), S3I 201 (STAT3 inhibitor), AG-490 (JAK2 selective inhibitor), and filgotinib (JAK-inhibitor) attenuated the profibrotic effects of PDGF highlighting the central role of the JAK-STAT pathway in the development of atrial fibrosis and AF in heart failure (Chen, 2017).

#### Micro-Ribonucleic Acids

Micro-ribonucleic acids (miRNAs) are 18- to 22-nucleotide RNA sequences capable of negatively modulating the expression of specific families of genes through the interaction with messenger RNAs (mRNAs) (Luo et al., 2015). The activity of specific miRNAs is involved in fibrotic responses associated with AF. MiR-30 and miR-133 interact with TGFβ and its receptor (Duisters, 2009; Chen, 2014). Moreover, miR-29, whose expression is reduced in the atria of HF patients, interacts with the mRNAs coding for collagen and fibronectin. MiR-26 is another player in the atrial fibrotic response. In AF, miR-26 is down-regulated by the activation of Ca^2+^/calmodulin/calcineurin signaling (Luo, 2013). MiR-26 is capable of targeting KCNJ2, the gene encoding for the I_K1_ (inward rectifier) K^+^ current. As a consequence, miR-26 down-regulation in AF promotes an increased expression of KCNJ2/I_K1_ in both fibroblasts (Qi, 2015) and cardiomyocytes (Luo, 2013). In fibroblast, increased I_K1_ hyperpolarizes the cell membrane, impairs atrial conduction (see above), and stimulates fibroblast activation. In cardiomyocytes, increased I_K1_ shortens action potential duration and atrial refractory period, thus stabilizing re-entrant rotors (Pandit, 2005).

**Figure 1:**
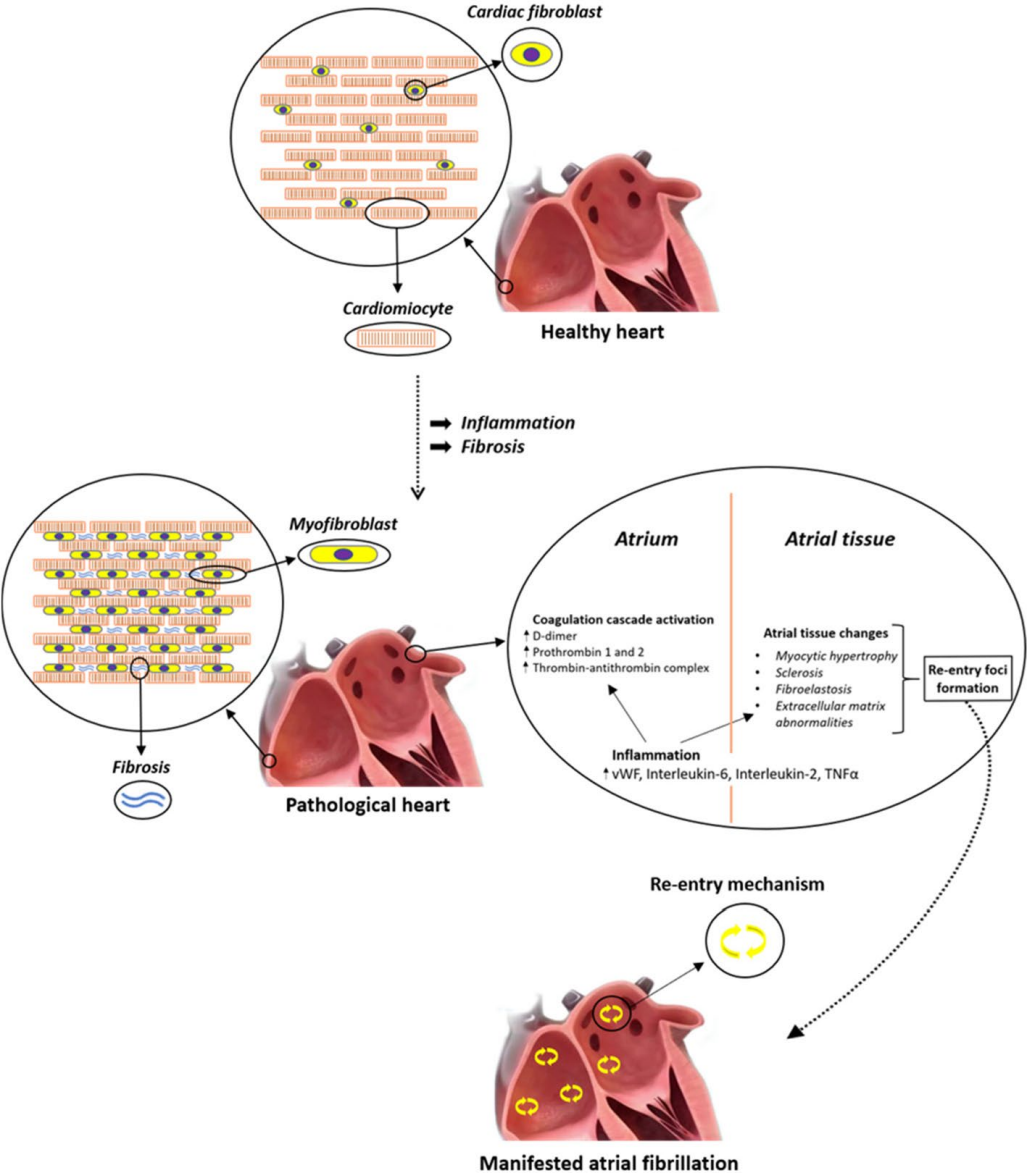
Systemic inflammation correlates with an increased risk of atrial fibrillation (AF) and thrombogenesis. Different inflammatory mediators, such as interleukin (IL)-2 and tumor necrosis factor alpha (TNFα), affect calcium handling and cause arrhythmogenic changes in atrial cardiomyocytes. In particular, altered TNFα-mediated Ca2+ handling could lead to arrhythmias due to calcium waves and delayed after-depolarization, which may generate premature APs and give birth to spontaneous beats that can then perpetuate themselves through re-entry mechanism. In addition, circulating cytokines can also stimulate NF-κB within atrial cardiomyocytes, leading to activation of the apoptosis cascade, which in turn is responsible for cardiomyocyte death and fibrous substitution.

The role of atrial inflammation and fibrosis in AF is summarized in **Figure 1**.

## Coagulation Factor Proteases and PARs in AF

**Figure 2:**
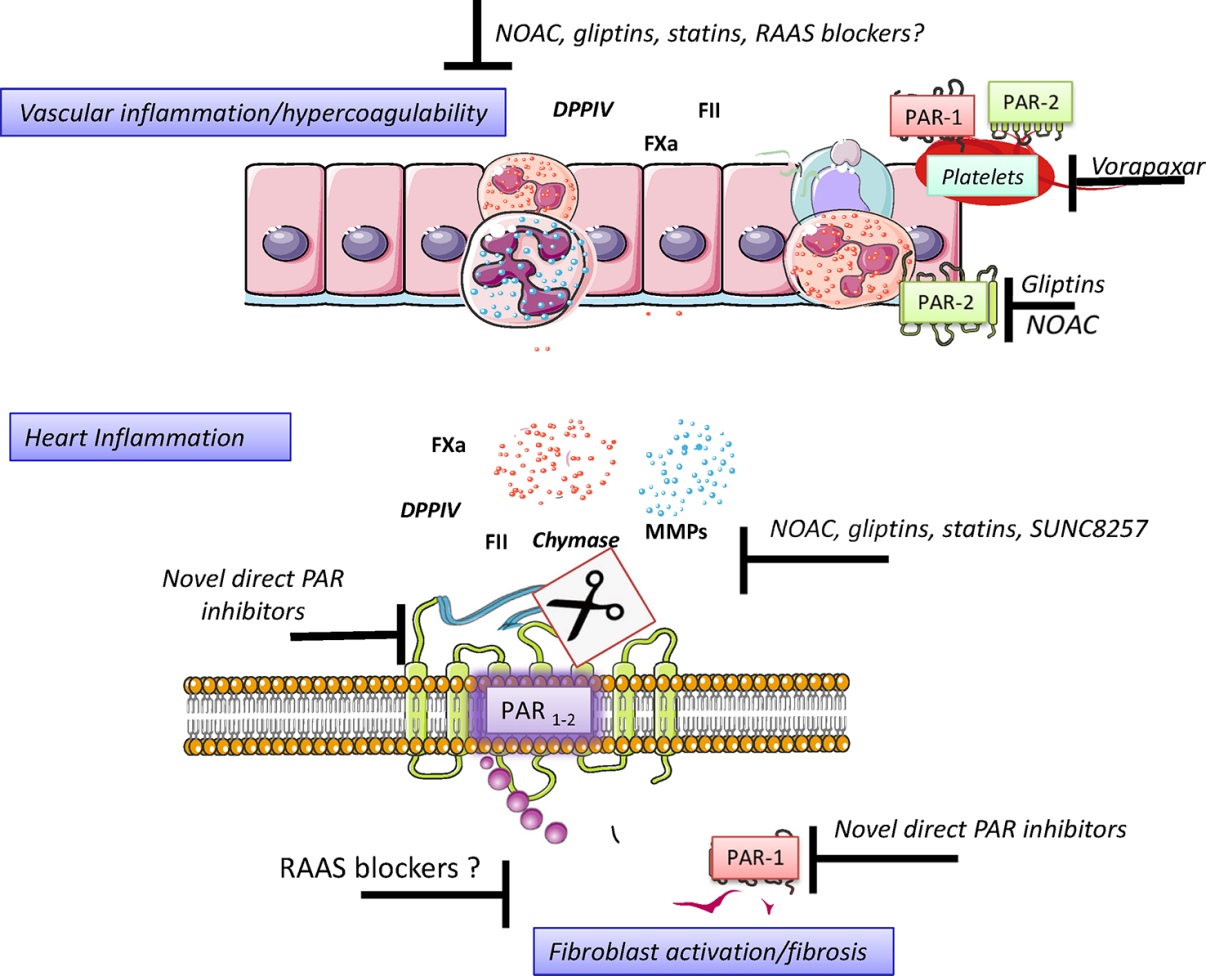
Serine proteases mechanisms of action. Serine proteases are a particular types of endoproteases involved in endogenous processes including the activation of inactive peptide (zymogens; i.e., coagulation cascade), inactivation of bioactive peptides (GLP-1 as DPP-4). Serine protease activity is controlled homeostatically by endogenous inhibitors (serpins). Furthermore, serine protease may also activate specific protease activated receptors (PAR) by cleaving the N-extracellular portion of the receptor and producing a tethered ligand. Such ligand interaction with the specific site of the trans-membrane-portion of the receptor induces G-protein recruitment and intracellular cascade including G-protein-dependent and -independent cascades. Figure created using Servier medical art templates.

**Figure 3:**
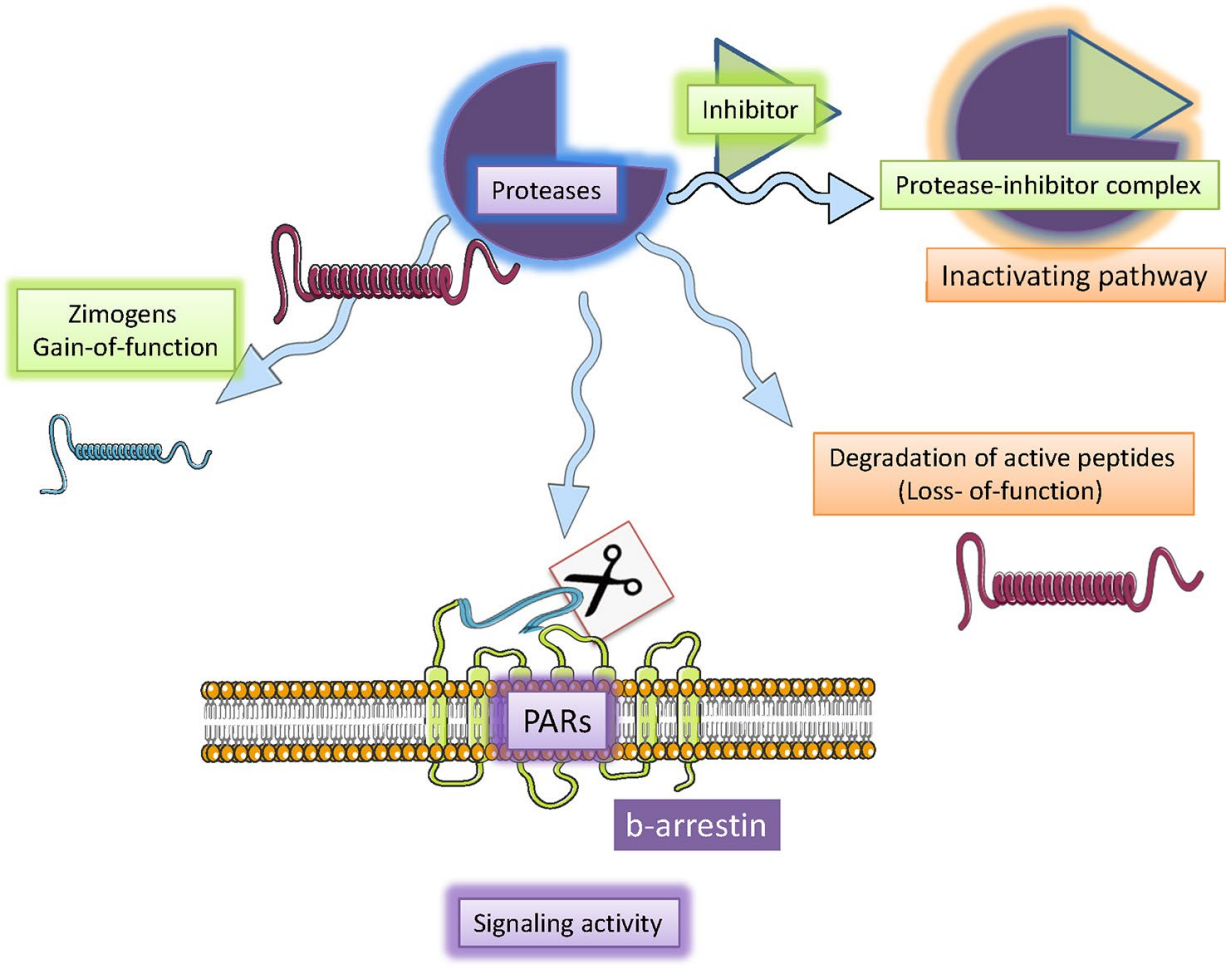
Direct anticoagulants may be thought as indirect PAR inhibitors. Direct anticoagulants including low molecular weight heparins and novel oral anticoagulants (NOAC) have different pharmacokinetic features including the volume of distribution and oral availability but share their ability to inhibit FII and FXa activity. Irrespective of their mechanism of coagulation factors inhibition, direct anticoagulants mimic serpin activity. This effect in turn reduces the possibility that coagulation factors may activate PAR and generate a pro-fibrotic cascade. Figure created using Servier medical art templates.

**Figure 4:**
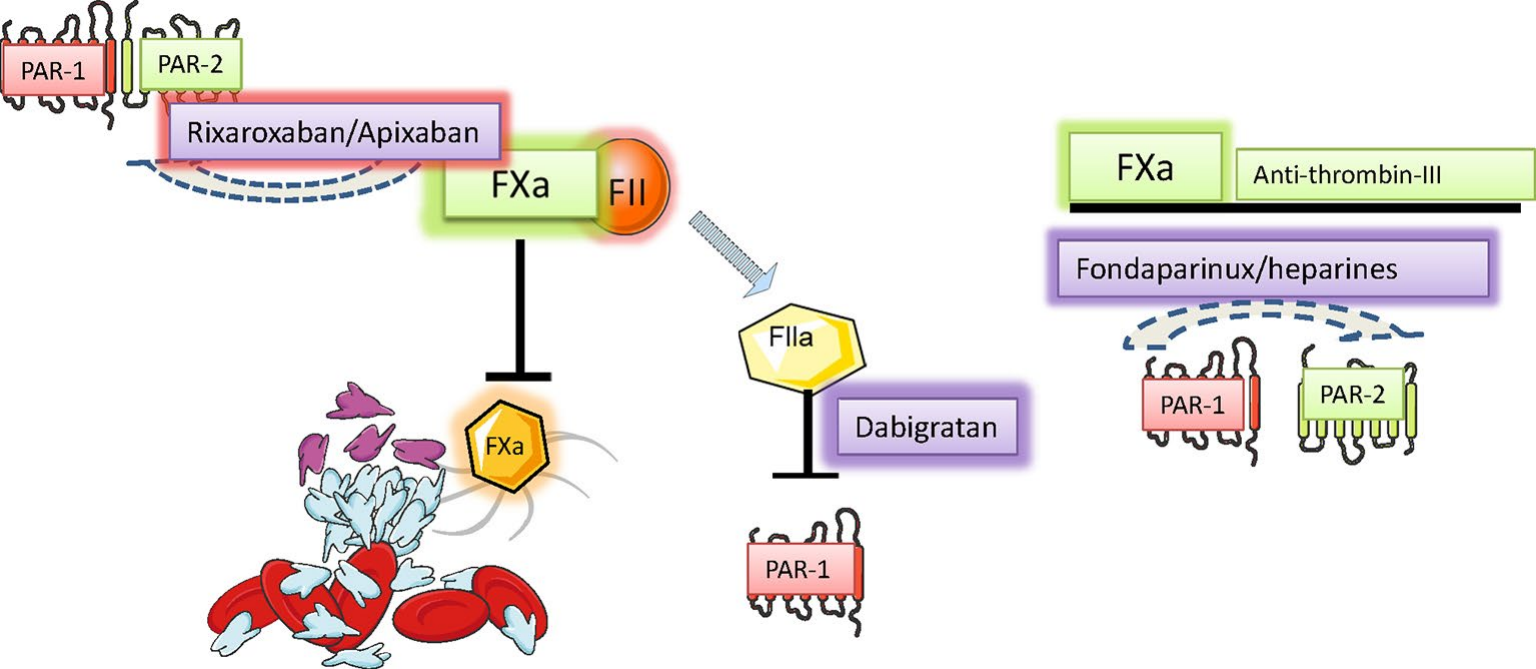
Pharmacological strategies to control hyperacoagulability and heart inflammation. Hypercoagulability generates and sustains vascular inflammation and permeability, a condition allowing the chemotaxis of inflammatory cells to neighbor tissues. Activated Inflammatory cells secrete, among the many signals, serine proteases, and disseminate inflammation in the invaded tissue. The control of serine protease activity may be achieved pharmacologically directly and indirectly by using drugs already in use or by promising novel drugs with proved effectiveness in preclinical investigations. Figure created using Servier medical art templates.

Atrial ﬁbrillation (AF) is responsible for the activation of blood coagulation (Watson et al., 2009), mainly due to blood stasis in the dilated fibrillating atria, which are unable to efficiently expel blood through contraction. Coagulation factor X is a vitamin K-dependent serine protease playing a central role in the cascade of blood coagulation, whose main activity is the conversion of prothrombin into thrombin (Borensztajn et al., 2008). Thrombin (factor II) is also a serine protease that converts fibrinogen into fibrin, thus forming blood clots (**Figure 2**) Recently, an additional role of activated factor X (FXa) and thrombin, non-related with coagulation, has been identified (Leadley et al., 2001): activation of PARs, PAR-1 and PAR-2. PAR-1 was identified as a rather selective receptor of thrombin (activated factor II) in 1991 and represents the first of an unique subclass of G-protein-linked seven-transmembrane domains receptors, that is, receptors activated by proteolytic cleavage of a N-terminal portion of their extracellular domain by specific proteases (Coughlin, 2000; Ossovskaya and Bunnett, 2004) (**Figure 3**). A reignited interest into the signaling cascades initiated by coagulation factors started to grow upon the identification of PAR-2 as a fundamental player in the development and progression of several disease states characterized by inflammation and fibrosis, including renal diseases (Grandaliano, 2003), cancer (Nierodzik and Karpatkin, 2006), abnormal wound healing (Diegelmann and Evans, 2004), as well as cardiovascular diseases such as atherosclerosis (Borissoff et al., 2011). PAR-1 and -2 receptors are expressed in many different tissues including the airways, the epidermis, the kidney, the intestine, the central nervous system, and the pancreas. In the cardiovascular system, they are expressed in endothelial cells, vascular smooth muscle cells, fibroblasts, and cardiomyocytes (Borensztajn et al., 2008). FXa preferentially activates PAR-2 when the protease is part of the ternary complex tissue factor (TF)–FVIIa–FXa, while lone FXa is active on both PAR-1 and PAR-2 with lower agonist activity(Ruf et al., 2003). PAR-1 and PAR-2 are associated with Gq proteins and thus, upon activation, stimulate phospholipase C to hydrolyze phosphatidylinositol into inositol tri-phosphate (IP3) and di-acyl glycerol (DAG). IP3 forces Ca^2+^ mobilization from the intracellular stores, while DAG activates protein-kinase C, which affects several downstream targets. Moreover, PAR receptors also activate extracellular-signal related kinase (ERK) and c-Jun N-terminal kinase (JNK) pathways, which lead to the initiation of a number of specific transcriptional programs (Borensztajn et al., 2008). In general, activation of these pathways stimulates cell growth and differentiation, production of cytokines, production and deposition of ECM components, and expression of adhesion molecules. In endothelial cells, PAR receptors stimulate production of adhesion molecules (E-selectin, VCAM), chemoattractant cytokines, and vascular permeabilization, thus promoting local inflammation and tissue infiltration (Ossovskaya and Bunnett, 2004). In fibroblasts, PAR activation causes cell proliferation, transition into myofibroblasts, production of collagen and fibronectin (Blanc-Brude, 2005), as well as production of chemoattractant cytokines (IL-6, IL-8, and monocyte chemoattractant protein-1 [MCP-1]) (Bachli, 2003). In cardiac fibroblasts, PAR-1 can transactivate epidermal growth factor receptor (EGFR), which in turn leads to activation of extracellular signal-regulated kinase, p38-mitogen-activated protein kinase, and protein kinase B (Sabri, 2002). The possible role of PARs in the development and progression of AF and atrial remodelling has been investigated in a few studies (**Figure 4**). Spronk et al. evaluated whether structural remodeling observable in the atria during AF could be stimulated by the hypercoagulable state. The authors worked on transgenic mice with a pro-coagulant phenotype (TMpro/pro mice), carrying a mutation on the thrombomodulin gene responsible for a reduced degradation of Factors V and VIII, observing an increased AF inducibility and stability, as well as enhanced collagen deposition (Spronk, 2017). These results support the potential involvement of hypercoagulability in the development of AF-related electrical and structural abnormalities. The authors of this work also found that administration of Nadroparin in goats with a hypercoagulable state associated to AF, mediating inhibition of Factor Xa-mediated thrombin generation, was able to partially affect the development of atrial ﬁbrosis (Spronk, 2017). These results suggest that anticoagulation therapy can partially attenuate atrial ﬁbrosis thus preventing the development of AF-related atrial abnormalities, though the authors did not demonstrate a direct mechanistic relationship between hypercoagulability and atrial fibrosis, and did not study PAR signaling in their AF models. A detailed study conducted on fibroblasts isolated from the atrial appendage of patients with AF who underwent cardiac surgery (Altieri, 2018a) revealed that atrial fibroblasts express high levels of PAR-1; when activated in vitro by thrombin-mediated cleavage, PAR-1 elicited myofibroblast transformation; production of collagen; and synthesis of TGFβ, MCP-1, and endothelin-1. Interestingly, dabigatran, an inhibitor of thrombin, prevented all these effects (Altieri, 2018b). The results of this work support the idea that thrombin inhibition may counteract the molecular and cellular events leading to the formation of AF-related functional and morphological changes in the diseased atria. Another elegant study was conducted on cultured tissue slices from human atrial biopsies (Bukowska, 2013). Atrial tissue was cultured in the presence of FXa and was subjected to rapid pacing to resemble AF. After 24 h, expression of PAR-1, PAR-2 and a number of inflammatory mediators such as IL-8 were found increased. These effects were inhibited the FXa inhibitor rivaroxaban and by the experimental PAR-2 inhibitor GB83 (Bukowska, 2013). In mice with atrial dilatation due to trans-aortic constriction, an increased expression of PAR-2 was observed in the atria, alongside an increased production of pro-inflammatory cytokines. Pre-incubation with the factor Xa inhibitor rivaroxaban suppressed these pathological changes (Kondo, 2018). In a rat model of heart failure associated with left atrial dilation, it was observed that direct thrombin inhibitors prevented left atrial dilatation, fibroblast activation, tissue fibrosis, reduced atrial tissue macrophage infiltration, and suppressed the inducibility of atrial arrhythmias with local electrical burst stimuli. Interestingly, none of these effects were observed with warfarin and similar results were obtained with the experimental inhibitor of PAR-1 F16618 (Jumeau, 2016).

Activation of PAR-1 in the atria favors AF not only because it stimulates fibrosis and inflammation but also due to its direct effects on the electrical activity of atrial cardiomyocytes. A study in isolated cardiomyocytes showed that thrombin-activated PAR1 induced a tetrodotoxin-sensitive late sodium current, which may lead to intracellular calcium overload and spontaneous arrhythmic activity; this effect was mediated by the calcium-independent phospholipase-A(2) signaling pathway (Pinet, 2008). In another study, PAR-1 activation increased intracellular cardiomyocyte [Ca^2+^] with membrane-independent mechanisms (Ide, 2007). A study investigated the effects of PAR-1 activation in isolated rabbit PV preparations: thrombin and blood-clot solutions increased left atrial diastolic tension, shortened action potential duration, and decreased atrial contractility; moreover, they led to depolarization of diastolic membrane potential, delayed after-depolarizations, and bursts of spontaneous activity. All these effects were prevented by the simultaneous application of BMS 200261, a PAR-1 inhibitor (Chang, 2012). Additionally, in the same model it was observed that the thrombin inhibitor dabigatran (Chang, 2012) and the FXa inhibitors edoxaban and rivaroxaban reduced the rate of spontaneous beating in the PV arrhythmogenic hotspot. This effect was mediated by inhibition of late Na^+^ current within atrial cardiomyocytes in the PV area and was mimicked by the application of BMS200261, a PAR-1 inhibitor (Chang, 2018). In another study, apixaban increased action potential duration and reduced spontaneous diastolic depolarization in rabbit PV preparations, an effect that was suppressed by the application of the PAR-1 agonist SFLLR-NH2, while warfarin failed to show any benefits (Font, 2018). All in all, these studies show that PAR activation by hypercoagulation in the atria may favor the onset and progression of AF via three concurrent mechanisms: promotion of inflammation, atrial fibrosis, and induction of an arrhythmogenic phenotype in atrial myocytes.

Direct inhibitors of PAR-1 have been developed and are available in the clinic as antiplatelet drugs for secondary prevention of myocardial infarction, ischemic stroke, and peripheral artery disease (Tricoci, 2012; Bonaca, 2014; Magnani, 2015; Ungar, 2018). The antiaggregant effect is achieved through inhibition of the thrombi-receptor (PAR-1) expressed on the membrane of platelets (Moon, 2018). Vorapaxar has been available for more than 6 years, and the results obtained in atherothrombotic diseases were rather modest in terms of reduction of new acute ischemic events, and the drugs increases the risk of bleeding and hemorrhagic stroke (Sharma, 2017). However, in an animal model of HFpEF, vorapaxar reduced cardiac fibrosis and inflammation (Friebel, 2019). Moreover, vorapaxar reduces endothelial activation, cytokine production, and systemic inflammation (Schoergenhofer, 2018). Whether PAR-1 direct inhibition by vorapaxar affects the development and progression of AF remains to be investigated, both experimentally and clinically. An overview of the currently available pharmacological agents acting on PAR signalling is provided in **Table 1**.

**Table 1:** Drugs acting on PAR signaling.

DRUG CLASS [references]	CLINICALLY AVAILABLE MOLECULES	MECHANISM	CLINICAL USE	ROLE IN AF
Heparins (Spronk, 2017)	Unfractionated heparins, low molecular weight heparins	Fxa and thrombin inhibition by binding to anti-thrombins	Parenteral anticoagulants	Reduced atrial fibrosis
Heparin derivatives (Spronk, 2017)	Fondaparinux (penta-saccharide)	FXa inhibition	Parenteral anticoagulant	Reduced atrial fibrosis
Direct thrombin inhibitors(Chang, 2012; Jumeau, 2016; Altieri, 2018a)	Dabigatran, ximelagatran	Thrombin inhibition	(New) oral anticoagulants	Lower atrial fibrosis, inflammation, AF inducibility
Factor Xa inhibitors(Chang, 2012; Bukowska, 2013; Font, 2018; Kondo, 2018)	Apixaban, rivaroxaban, edoxaban	Reduction of thrombin formation	(New) oral anticoagulants	Lower atrial fibrosis, inflammation, firing of pulmonary veins, AF inducibility
PAR-1 inhibitors (Chang, 2012; Tricoci, 2012; Bonaca, 2014; Magnani, 2015; Jumeau, 2016; Chang, 2018; Schoergenhofer, 2018; Ungar, 2018; Friebel, 2019)	Vorapaxar	Block of PAR-1 signaling	Oral anti-platelet agent	Unknown
DPP-4 inhibitors (Lendeckel, 2001; Tremblay, 2014; Wronkowitz, 2014; Gong et al., 2015; Yamamoto, 2015; Chang, 2017; Zhang, 2017; Igarashi, 2018)	Sitagliptin, saxagliptin and other gliptins	Inhibition of circulating and local DPP-4, a PAR activator	Type-II diabetes (oral glucose lowering)	Reduced inflammation, small evidence
Chymase inhibitors (Matsumoto, 2003; Hooshdaran, 2017)	None	Inhibition of local chymases, PAR-1 activators	Experimental drugs	Unknown

## Role of Other Serine-Proteases in AF: Mast Cell Chymase, Dipeptidyl-Peptidase-4 and Prostate-Specific Antigen Kallikrein

We previously highlighted the possible role of mast cell proliferation in the onset and progression of AF. Interestingly, mast cells are the main cell type expressing chymase (Urata, 1993), a serine protease stored in mast cell granules, which acts on several targets that are relevant for atrial function and dysfunction. In particular, chymase can locally convert angiotensin-I to the active mediator angiotensin-II (Ang II), which notably promotes inflammation and fibrosis (see above). The presence of chymase in the heart can therefore limit the efficacy of ACE inhibitors (Wei, 2010). Indeed, in atrial samples from patients with AF who underwent surgery, it was observed that chymase mediated the formation of Ang II from angiotensin-(1-12) (Ahmad, 2011). It remains to be established whether mast cell chymase can directly activate PAR receptors in cardiac tissue. The experimental chymase inhibitor SUNC8257 partially prevented ventricular remodeling in an animal model of tachycardia-induced heart failure, leading to reduction of diastolic dysfunction and atrial dilatation (Matsumoto, 2003). In a model of cardiac infarction, chymase inhibition prevented myocyte apoptosis and fibroblast migration/differentiation (Hooshdaran, 2017). In different animal models of myocardial infarction, treatment with specific inhibitors of mast-cell chymase, including TEI-E548, BCEAB, and others, led to reduction of hypertrophy, improvement of diastolic function, reduced arrhythmias, and prolonged survival (Jin, 2002).

Whether chymase inhibition represents a feasible strategy to prevent atrial remodeling in the pathogenesis of AF remains to be investigated.

Dipeptidyl-peptidase-4 (DPP4) is a serine protease expressed in several cell types and organs, which is known to cleave and inactivate the incretin glucagon-like peptide-1 (GLP-1). In the heart, DPP4 is mainly expressed in the membrane of the capillary endothelium, in the sarcolemma of cardiomyocytes and is also produced by infiltrating macrophages (Kobara, 2018; Gorrell, 2005). Oral DPP4 inhibitors (gliptins) are used in type-2 diabetes to potentiate GLP-1 signaling. A recent study observed that in obesity, DPP4 secreted by the liver is able to activate PAR-2 receptors expressed by macrophages, in particular macrophages that populate adipose tissue, which in turn are activated and promote a generalized pro-inflammatory state (Ghorpade, 2018). Moreover, soluble DPP4 can mediate endothelial dysfunction, acting via activation of PAR-2 and consequent release of prostanoids with vasoconstrictor properties (Romacho, 2016). Also, soluble DPP-4 can activate PAR-2 in vascular smooth muscle cells, leading to production of pro-inflammatory cytokines (Wronkowitz, 2014). Recent work suggested that circulating and/or locally expressed DPP4 plays a role in AF pathogenesis (Lendeckel, 2001; Yamamoto, 2015; Chang, 2017; Zhang, 2017; Igarashi, 2018). A recent observational study revealed that diabetic patients treated with DPP4 inhibitors (gliptins) had a lower risk of AF as compared with patients treated with other drugs (Chang, 2017). Ectopeptidases such as DPP-4 are expressed in human atrial biopsies from surgical patients, independently from the presence or absence of AF (Lendeckel, 2001). In a canine model of AF due to rapid atrial pacing, linagliptin partially prevented the shortening of atrial refractory period and the slowing of atrial conduction induced by rapid pacing, thus lowering AF inducibility (Igarashi, 2018). In a rabbit heart failure model (ventricular tachypacing), treatment with alogliptin reduced atrial fibrosis and increased atrial capillary density, ultimately reducing the duration of AF episodes (Yamamoto, 2015). In diabetic rabbits showing atrial dilatation, fibrosis, and elevated AF propensity, treatment with alogliptin partially prevented atrial abnormalities, thanks to the reduction of mitochondrial ROS production by atrial cardiomyocytes (Zhang, 2017). However, whether these positive effects of DPP-4 inhibitors on atrial myocardium function depend on the reduced PAR activation remains to be studied. Other mechanisms, involving the angiotensin-II/sodium-proton pump exchanger-1 axis, have been suggested as mediators of the direct cardiac effects of these agents. In addition, gliptins were shown to have a clear anti-inflammatory effect, leading to decreased circulating pro-inflammatory cytokines (Gong et al., 2015) and endothelial expression of adhesion molecules (Tremblay, 2014); in line with this, while high-fructose/glucose diet increases the expression of TGFβ and MCP-1 in the myocardium, treatment with DPP-4 inhibitors reduced these cardiac inflammation markers (Bostick, 2014). However, it is unclear whether these anti-inflammatory effects of gliptins are mediated by the reduced activation of PARs in the heart. All in all, gliptins have a clear favorable profile in AF and may reduce AF-related atrial structural and electrical changes. Unfortunately, in two large clinical trials with DPP4 inhibitors (Scirica, 2013; White, 2013), no significant improvements in hard cardiovascular endpoints were noted, albeit no specific analysis on the rate of AF incidence was conducted. It is to be noted that in all studies performed in type-2 diabetes patients gliptins were used on top of metformin. Of note, metformin treatment was shown to reduce the risk of AF in population studies (Chang, 2014). Therefore, the beneficial effects of gliptins on AF may, at least in part, depend on their synergic interaction with metformin. Moreover, GLP-1 is increased by DPP-4 inhibitors and the potentiation of GLP-1 dependent signaling may be involved in the suppression of atrial remodeling by gliptins. Indeed, the GLP-1 analog liraglutide suppressed most of the atrial electrophysiological changes induced by rapid atrial pacing in a dog model, ultimately reducing the inducibility of AF (Nakamura, 2019). However, a meta-analysis of several clinical studies with GLP-1 analogs in diabetic patients showed that they were not associated with a reduced incidence of AF (Monami, 2017).

Tissue kallikreins are a family of serine proteases that are expressed in several tissues and organs throughout the body (Wu et al., 2005), though their levels are relatively low in cardiac tissue (Nolly, 1994). Their main function is the conversion of kininogen to bradykinin, a potent vasoactive agent that is involved in vasodilation, pain, and inflammation (Bhoola et al., 1992), but may also convert Ang I to Ang II (Arakawa and Maruta, 1980). Reduced urinary excretion of tissue kallikreins has been associated with hypertension, in line with the anti-hypertensive effects of the kallikrein-kinin system activation (Margolius, 1974). Moreover, kallikrein levels in atrial tissue are increased in patients type II diabetes mellitus (Campbell, 2010), suggesting that this cardioprotective system may be active in these patients to mitigate the deleterious effects of hyperglycemia in the heart. Kallikreins showed clear cardio-protective effects in animal models of myocardial infarction (Yoshida, 2000). Interestingly, kallikrein-deficient transgenic mice develop a severe dilated cardiomyopathy (Meneton, 2001), which does not seem to be a consequence of the reduced bradykinin activation (Pesquero, 2000), suggesting additional physiological roles of kallikreins in the heart. Prostate-specific antigen (PSA), also known as gamma-seminoprotein or kallikrein-3 (KLK3), is a glycoprotein enzyme encoded in humans by the KLK3 gene and secreted by the epithelial cells of the prostate gland. Its physiological role is the liquefaction of the semen to facilitate sperm movement, but it may play a role in the pathophysiology of cardiovascular diseases(Patane and Marte, 2009). Indeed, increased serum PSA has been reported in patients who received cardiopulmonary resuscitation(Koller-Strametz, 2000), invasive open-chest cardiac surgery(Mahfouz, 2008), and in those who suffered from acute myocardial infarction(Crook et al., 1997). Interestingly, patients with myocardial infarction who had a marked elevation of PSA showed an increased likelihood of paroxysmal AF in the first days after the event(Patane and Marte, 2010a; Patane and Marte, 2010b). Moreover, high PSA levels correlated with the risk of new-onset AF in hypertensive patients (Patane and Marte, 2012). However, it is unclear from these studies whether PSA plays an active role in the pathogenesis of AF and myocardial infarction or whether it is only an epiphenomenon, that is, PSA elevation goes in parallel with systemic inflammation levels. In contract with the previous observations, some studies go against the idea that PSA or other kallikreins play an active role in the pathophysiology of AF: infusion of the general kallikrein/serine protease inhibitor aprotinin was not associated with a significant reduction in post-operative AF in patients undergoing cardiothoracic surgery (Gillespie, 2005).

## Summary and Conclusion

AF is the most common cardiac arrhythmia and the main risk factor for ischemic stroke due to the associated hypercoagulability leading to arterial embolism. Though a number of comorbidities are recognized as risk factors for AF (hypertension, CAD, diabetes obesity), the mechanisms leading to the onset of AF and to its chronicization remains to be clarified. Overall, clinical and experimental data indicate that AF is a multifactorial disease whose pathogenic ground might be the presence of a low-grade systemic and/or local inflammation. In this context, the search for novel drug targets remains a primary issue to help reducing inflammation, in order to avoid the development of fibrosis.

Clinical observational studies indicate that AF is associated with increased levels of systemic inflammatory marker. Episodic AF is able to generate atrial inflammation, inflammatory cells infiltration, and an increase of atrial macrophage population; the activation of infiltrating inflammatory cells, by the mean of their secretome, triggers stable pro-fibrotic structural changes within atrial cardiomyocytes and all the surrounding cell types, thus contributing to generate stable atrial structural changes that alter atrial electrical conduction and stabilize arrhythmogenic reentry circuits. In this respect, a central role is likely to be played by fibroblasts, which, upon activation by different pathways such as the renin angiotensin system, embrace the surviving atrial cardiomyocytes in a sheath of fibrous material, with severe arrhythymogenic consequences. Overall, experimental evidence suggests to monitor and detect early signs of low-grade atrial inflammation, before it evolves toward atrial fibrosis.

Pharmacological therapy of AF includes anticoagulants and long-term therapy with anti-fibrotic drugs, such as statins and renin/angiotensin system blockers, including aldosterone receptor antagonists. These drugs demonstrated a certain effectiveness in reducing atrial inflammation and fibrosis and showed some anti-arrhythmic potential in several clinical settings.

In apparent contrast with the inflammatory hypothesis, the clinical effectiveness of classical anti-inflammatory drugs in AF [i.e., corticosteroids or non-steroideal anti-inflammatory drugs (NSAIDS)] remains elusive. However, this discrepancy likely derives from the heterogeneity of the clinical data available and the lack of focused studies. Overall, pharmacological data confirm the benefits of treating patients with well-tolerated molecules that oppose the effects of pro-hypertrophic and pro-fibrotic signals and that the timing of therapy initiation is a main determinant of its eventual success.

Serine proteases are among the main players of inflammation. These enzymes can be found circulating in blood (e.g., coagulation factors), but are also components of the proteolytic capacity of all the cell types that form the cardiovascular system. Serine proteases are also found in the secretome of activated inflammatory cells; these proteases play a fundamental role in controlling extracellular matrix production/degradation. Physiologically, serine proteases participate in the activation/deactivation of signaling peptides and play critical roles in regulating several homeostatic functions. In these sense, the most relevant proteases are DPP4, coagulation factors, and mast cells chymase. All these enzymes have signaling activities in addition to their biological action; this additional action is accomplished by activating specific G-protein coupled receptors, namely PARs, of which four isoforms are known (PAR1-4). Serine proteases activate PARs promoting the formation of an N-tethered ligand, which triggers the initiation of an intracellular cascade, a mechanism becoming relevant when protease levels overcome the buffering capacity of their endogenous inhibitors. PARs show complex mechanisms of activation (transactivation and formation of homo and etherodimers) and deactivation, may be present in biased conformations or activated by biased agonists. PAR expression/activity is increased in the atria and in the coronary vessels of AF subjects. Overall, experimental evidence indicates that the control of PAR signaling by serine proteases might participate to cardiac fibrosis and then considered a novel target for drug activity in AF. A direct PAR-1 inhibitor is available in the clinic. Vorapaxar is the first PAR1 inhibitor indicated as a novel antiplatelet drug, since PAR-1 is crucial for thrombin-induced platelet aggregation. Despite clinical evidence indicated that vorapaxar administration is associated with a reduction of cardiac fibrosis, the clinical pharmacological profile of vorapaxar is complicated by the elevated incidence of adverse events, including severe bleeding. These conflicting results confirm that PAR pharmacological inhibitors should be managed carefully. Alternatively, a different and safer approach might be to block PAR indirectly by using serine-protease inhibitors, such as DPP4 and chymase inhibitors, or direct anticoagulants.

Gliptins are inhibitors of soluble and tissue-bound DPP4 that exert their antidiabetic action by increasing incretin hormone levels. Interestingly, DPP4 is expressed in all cell cardiac types and plays an important role in vascular endothelium and on cardiac fibroblasts, where it activates PAR2. Because of this, gliptins potentially reduce the capacity of DPP4 to activate PAR2, with a beneficial effect on endothelial dysfunction, the main cellular mechanism predisposing to inflammation, typically occurring in the diabetic vasculature. Through PAR inhibition, gliptins may reduce the pro-fibrotic cascade triggered by PAR2 activation. In addition, gliptins have the potential to increase the levels of cardiac protective hormones, such as GLP-1. Clinical evidence indicates that only aloglitpin, a third-generation gliptin, is effective in reducing AF chronicization, confirming that the cardiovascular protection of gliptins is molecule-dependent and may not be a class effects. However, the demonstrated effectiveness of alogliptin highlights the possibility to use gliptins also in not diabetic patients, given the excellent safety profile of these drugs.

At the moment, chymase inhibitors are still at the preclinical level of investigation. However, even if much remains to be investigated in respect of the most promising molecules, experimental evidence indicates their effectiveness in targeting and reducing cardiac fibroblast activation.

Coagulation factors, including thrombin and FXa are activators of PAR1 and PAR1 and PAR2, respectively. Experimental evidence suggests that increased levels of coagulation factors may also have a direct role in the onset of cardiac fibrosis. Interestingly, thrombin activation of PAR-1 in the atria, whose expression is increased in AF, generates pro-fibrotic pathways and affects the electrical activity of atrial cardiomyocytes. Since hypercoagulability is a risk factor for ischemic stroke in AF patients, anticoagulants are very often prescribed in AF patients.

Direct anticoagulants, low molecular weight heparins, and oral anticoagulants target the enzyme activity of thrombin and of FXa, thus reducing coagulation but also their probability to activate the PAR1 and PAR2 receptors that are expressed in the cardiovascular system. Experimental evidence indicates that heparins, thrombin inhibitors, and FXa inhibitors reduce atrial remodeling and fibrosis in different experimental settings. Heparins and oral anticoagulants have different pharmacokinetic features; the fact that direct oral anticoagulants are as effective as heparins in reducing atrial fibrosis and arrhythmogenenicity suggests that the beneficial effects of direct anticoagulants are mostly produced at the vascular level, as heparins cannot penetrate into tissues. No advantage of using FXa versus thrombin inhibitors as protective agents in AF are documented.

Taken together, this evidence suggests that the use of direct anticoagulants and, in general, of serine proteases inhibitors, including gliptins and possibly chymase inhibitors, might be included within the armamentarium of the pharmacological treatment for AF. However, only appropriately designed clinical trials will tell us whether these drugs really behave as disease modifying agents in this disease.

## Author Contributions

All the authors participated in collecting and discussing the literature data. RC, LoS and LR drafted the manuscript and the figures. EC and LaS critically reviewed the manuscript and edited the figures.

## Funding

This article was supported by a local grant from the University of Florence (Università degli Studi di Firenze), by Ente Cassa di Risparmio di Firenze and by the *Italian Ministry of Education, University, and Research* (MIUR PRIN 2018 grant RHYTHM-Insight to EC).

## Conflict of Interest

The authors declare that the research was conducted in the absence of any commercial or financial relationships that could be construed as a potential conflict of interest.
